# Altered Peroxisome Proliferator-Activated Receptor Alpha Signaling in Variably Diseased Peripheral Arterial Segments

**DOI:** 10.3389/fcvm.2022.834199

**Published:** 2022-06-15

**Authors:** Connor Engel, Rodrigo Meade, Nikolai Harroun, Amanda Penrose, Mehreen Shafqat, Xiaohua Jin, Gayan DeSilva, Clay Semenkovich, Mohamed Zayed

**Affiliations:** ^1^Section of Vascular Surgery, Department of Surgery, Washington University in St. Louis School of Medicine, St. Louis, MO, United States; ^2^Division of Molecular Cell Biology, Washington University in St. Louis School of Medicine, St. Louis, MO, United States; ^3^Department of Biomedical Engineering, McKelvey School of Engineering, Washington University in St. Louis, St. Louis, MO, United States; ^4^Veterans Affairs St. Louis Health Care System, St. Louis, MO, United States; ^5^Division of Endocrinology, Metabolism and Lipid Research, Department of Medicine, Washington University in St. Louis School of Medicine, St. Louis, MO, United States

**Keywords:** PPARα, *acox1*, *cpt1a*, peripheral arterial disease, atherosclerosis, diabetes

## Abstract

**Objective:**

Peripheral atherosclerosis that accumulates in the extracranial carotid and lower extremity arteries can lead to significant morbidity and mortality. However, atherosclerotic disease progression is often not homogenous and is accelerated by diabetes. We previously observed increased phospholipid content in minimally (Min)-diseased arterial segments compared to maximally (Max)-diseased segments. Since Peroxisome Proliferator-Activated Receptor alpha (PPARα) is a key regulator of lipid metabolism, we hypothesized that it may have differential expression and signaling in Min vs. Max-diseased peripheral arterial segments.

**Methods:**

Eighteen patients who underwent carotid endarterectomy (CEA), and 34 patients who underwent major lower extremity amputation were prospectively enrolled into a vascular tissue biobank. Min and Max-diseased segments were obtained in real-time from CEA plaque and amputated lower extremity arterial segments. mRNA and protein were isolated from specimens and the relative expression of *ppara*, and its downstream genes Acyl-CoA Oxidase 1 (*acox1*) and Carnitine Palmitoyltransferase 1A (*cpt1a*) were also evaluated. We evaluated gene expression and protein content relative to atherosclerotic disease severity and clinical diabetes status. Gene expression was also evaluated relative to Hemoglobin A1c and serum lipid profiles.

**Results:**

In CEA segments of patients with diabetes, we observed significantly higher *ppara* and *acox1* gene expression (*p* < 0.01 and *p* < 0.001 respectively), and higher PPARα protein content (*p* < 0.05). Hemoglobin A1c significantly correlated with expression of *ppara* (R^2^ = 0.66, *p* < 0.001), *acox1* (R^2^ = 0.31, *p* < 0.05), and *cpt1a* (R^2^ = 0.4, *p* < 0.05). There was no significant difference in gene expression between Min vs. Max-diseased CEA plaque segments. Conversely, in lower extremity arterial segments of patients with diabetes, we observed significantly lower *ppara*, *acox1*, and *cpt1a* expression (*p* < 0.05, *p* < 0.001, and *p* < 0.0001 respectively). Interestingly, CPT1A content was lower in arterial segments of patients with diabetes (*p* < 0.05). Hemoglobin A1c and HDL-cholesterol had negative correlations with *ppara* (R^2^ = 0.44, *p* < 0.05; R^2^ = 0.42, *p* < 0.05; respectively).

**Conclusion:**

This study demonstrates the significant differential expression of *ppara* and its immediate downstream genes in human carotid and lower extremity arteries relative to disease severity and diabetes. These findings highlight that mechanisms that influence atheroprogression in the carotid and lower extremities peripheral arteries are not homogenous and can be impacted by patient diabetes status and serum cholesterol profiles. Further elucidating these differential molecular mechanisms can help improve targeted therapy of atherosclerosis in different peripheral arterial beds.

## Introduction

**FIGURE 1:**
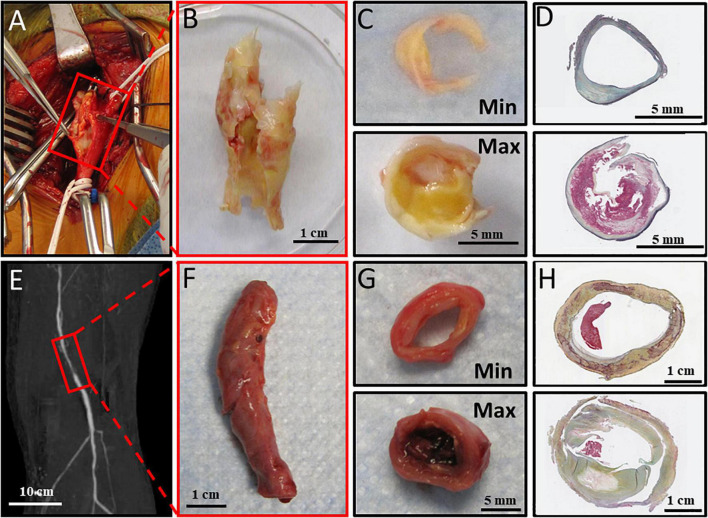
Atheroprogression of carotid artery bifurcation and lower extremity. **(A)** Carotid bulb before endarterectomy, **(B)** Carotid endarterectomy harvested from operating room, **(C)** Carotid Endarterectomy sectioned into Min and Max diseased sections. **(D)** Carotid endarterectomy Min and maximally Max sections with Movat Pentachrome staining. **(E)** Preoperative imaging showing reduced perfusion in the popliteal artery. **(F)** Dissected popliteal artery collected immediately from operating room. **(G)** Anterior tibial artery sectioned into minimally (Min) and maximally (Max) diseased components. **(H)** Min and Max sections stained with Movat Pentachrome staining. Movat pentachrome stains elastic fibers black, Nuclei Blue, Collagen Yellow, reticular fibers yellow, mucin bright blue, and fibrin red.

**FIGURE 2:**
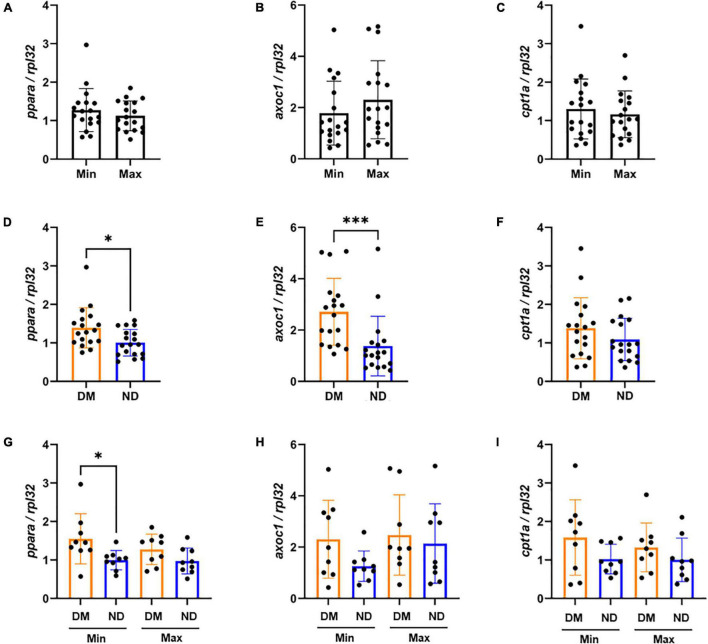
mRNA expression variability of carotid endarterectomy plaques by lesion severity and diabetic status **(A–C)** No significant difference in carotid artery plaque expression between the Min (*n* = 18) and Max (*n* = 18) segments of *ppara*, *acox1*, and *cpt1a* mRNA expression. **(D–F)** Combined Min and Max analysis of patients with diabetes (*n* = 18) demonstrate significantly higher mRNA expression of *ppara* (*p* < 0.05), and *acox1* (*p* < 0.001) compared to patients without diabetes. We did not observe a difference between *cpt1a* mRNA expression between patients with or without diabetes. **(G–I)** When distinguishing both disease severity and diabetic status, we found significant increase in *ppara* mRNA expression in patients with diabetes (*n* = 9) compared to patients without diabetes (*n* = 9). We found no significant differences in *acox1* or *cpt1a* expression. DM, Diabetic. ND, Non-diabetic. Min, Minimally diseased. Max, Maximally diseased. Data are presented as mean ± SD, and each point represents one patient sample. *p < 0.05 and ***p < 0.001.

Atheroprogression progresses at different rates in the peripheral arterial system—with areas that are more likely to develop a high burden of disease and others that are often rarely impacted (Figure 1; 1, 2). The extracranial carotid arteries and lower leg popliteal-tibial arterial segments are especially prone to atheroprogression and can lead to significant clinical morbidity (3–5). Approximately 20–30% of ischemic strokes that occur each year result from advanced atherosclerotic plaque formation in the carotid arteries (6–9). Similarly, in the lower extremities, nearly one million Americans will develop symptomatic lower extremity intermittent claudication each year, of which, 20% are estimated to progress to Chronic Limb-Threatening Ischemia (CLTI) (4, 9, 10). Patients with diabetes (11, 12) and hyperlipidemia (13, 14) are especially prone to carotid and popliteal-tibial arterial atheroprogression. Although significant strides have been made in the last two decades to decrease the morbidity associated atherosclerosis in these arterial beds, the continued high prevalence of disease necessitates continued investigation of the differential metabolic mechanisms that influence disease progression.

Peroxisome proliferator-activated receptor-alpha (PPARα) is an important transcription factor that in the liver regulates expression of enzymes required for β-fatty acid oxidation. These enzymes influence production of serum triglycerides and total serum cholesterol (15–17). In arterial tissue, PPARα is known to regulate arterial wall inflammation, lipid metabolism, and even atherosclerotic plaque formation (18–21). Our group recently demonstrated that choline-ethanolamine phosphotransferase 1 (*cept1*), an upstream regulator of PPARα, had increased gene expression in minimally (Min)-diseased peripheral tibial arterial segments in patients with diabetes (22, 23). Increased *cept1* gene expression in the peripheral arteries of individuals with diabetes led us to hypothesize that PPARα and its downstream effectors, Acyl-CoA Oxidase 1 (*acox1*) and Carnitine Palmitoyltransferase 1A (*cpt1a*), may also be increased in the setting of diabetes and vary relative to atherosclerotic disease severity. To evaluate this, we performed a novel assessment of Min vs. Max-diseased peripheral arterial segments that were harvested in real-time from patients who underwent carotid endarterectomy (CEA) or lower extremity amputation. The relative content of PPARα and its downstream genes were then correlated with arterial disease severity and patient diabetes status.

## Materials and Methods

### Human Subjects and Vascular Tissue Biobank

Between August 2015 and February, 2020, 52 patients underwent elective CEA or major lower extremity amputation and were prospectively enrolled to participate in an institutional review board-approved human vascular tissue biobank. All recruited patients provided informed consent for harvesting of intra-operative CEA plaque or lower extremity popliteal-tibial arterial segments from the amputated limb. All harvested specimens were de-identified and cataloged in a vascular tissue biobank for further analysis.

### Patient Demographics

Patient demographics were collected via chart review and maintained in a de-identified secure patient database. These demographics were collected by chart review, and included sex, age, body mass index (BMI), smoking status, and medical history of diabetes (defined as a previous diagnosis of diabetes), hemoglobin A1c, fasting glucose, hypertension (defined as a systolic pressure ≥ 140 mmHg and/or a diastolic pressure ≥ 90 mmHg), hyperlipidemia (cholesterol levels ≥240 mg/dl, triglyceride levels ≥ 200 mg/dl, or LDL levels ≥ 160 mg/dl) (24; Table 1). Fasting glucose and hemoglobin A1c levels were measured the day of surgery, and lipid panels within 6 months of the operative date were also reviewed for analysis. Diabetes severity was evaluated using a Diabetes Clinical Severity Index (DCSI) as previously described (25), which is scored by compiling a patient’s history of diabetes-related complications and range from 0 to 13. Patients with a DCSI score above the mean in each surgical group were categorized as “High DCSI” while patients below the mean were categorized as “Low DCSI.”

**TABLE 1:** Patient clinical demographics.

	All Patients	CEA Patients	Amputation Patients	CEA vs. Amputation	NDM	DM	NDM vs. DM
Demographics	*n* = 52	*n* = 18	*n* = 34	*p*	26	*n* = 26	*p*
Sex (male: female)	32:20:00	11:7	21:13	1	15:11	17:09	0.78
Mean age (years) ± SD	63.4 ± 10.0	66 ± 9.3	62.1 ± 10.2	0.23	63.2 ± 9.4	63 ± 10.7	0.59
Mean BMI (kg/m^2^) ± SD	28.5 ± 8.3	29.1 ± 7.9	28.2 ± 8.7	0.23	26.7 ± 7.0	30 ± 9.3	0.23
**Co-morbid conditions**							
Diabetes mellitus (% of total)	26 (45.61%)	9 (50%)	17 (50%)	1	0	26 (100%)	n/a
Insulin Dependent (% of Diabetics)	7 (26.9%)	0 (0%)	7 (41%)	0.27	0	7 (27.0%)	n/a
Tobacco smoker (% of total)	44 (85%)	15 (84.6%)	29 (85.3%)	1	22 (84.5%)	22 (84.5%)	1
Hyperlipidemia (% of total)	39 (75%)	16 (88.9%)	23 (67.6%)	0.17	18 (69.2.0%)	21 (80.1%)	0.52
Mean DCSI ± SD	4.9 ± 2.1	4.1 ± 1.85	5.4 ± 2.2	0.21	N/A	4.9 ± 2.2	n/a
High DCSI (% of Diabetics)	N/A*	5 (55.6%)	7 (41%)	N/A*	N/A*	N/A*	n/a
Symptomatic Carotid Disease (% of CEA)	N/A	4 (44.4%)	N/A	N/A	2 (22.2%)	4 (44.4%)	0.67
Receiving Fenofribrate (% Total)	4 (7.7%)	3 (16.7%)	1 (2.9%)	28.3	1 (3.8%)	3 (11.5%)	0.61
Receiving Statin (% of total)	35 (67.7%)	13 (72.2%)	22 (64.7%)	0.76	14 (53.8%)	21 (80.7%)	0.07
Hypertension (% of total)	44 (84.6%)	16 (88.9%)	28 (82.3%)	1	22 (85.6%)	22 (84.6%)	1
**Anthropometric characteristics**							
HbA1c (mg/dL) ± SD	7.1 ± 1.8	6.8 ± 1.6	7.1 ± 2.0	0.55	5.7 ± 1.4	7.9 ± 1.7	**0.0002**
Fasting Glucose (mg/dL) ± SD	129.3 ± 41.1	135.2 ± 44.7	124.8 ± 38.7	0.69	103.5 ± 19.6	147 ± 43.8	**0.007**
Triglycerides (mg/dL) ± SD	149.5 ± 47.3	137.8 ± 76.8	144.1 ± 84.4	0.83	115.2 ± 66.8	159.8 ± 85.9	0.07
Total Cholesterol (mg/dL) ± SD	149.5 ± 46.8	172.1 ± 44.1	133.5 ± 44.0	**0.02**	149 ± 41.9	148 ± 51.8	0.66
LDL Cholesterol (mg/dL) ± SD	77.0 ± 37.0	95.5 ± 32.9	65.0 ± 35.3	**0.01**	84.1 ± 36.3	72.4 ± 36.4	0.47
HDL Cholesterol (mg/dL) ± SD	38.4 ± 13.0	40.2 ± 12.2	38.7 ± 13.8	0.64	39.7 ± 9.8	37 ± 15.054	0.58
Non-HDL Cholesterol (mg/dL) ± SD	119.8 ± 45.6	132 ± 47.75	96.3 ± 38.7	**0.04**	110.6 ± 43.2	110 ± 48.24	1

*Mean DCSI was calculated for each surgical group. DCSI, diabetes clinical severity index; HbA1c, hemoglobin A1c.

### Human Tissue Processing

For CEA specimens, plaque was immediately transferred to the research laboratory in cold saline on ice. Specimens were sectioned into Min and Max-diseased segments as previously described (26). Approximately 5 mm sections of tissue at the carotid bifurcation demonstrating American Heart Association (AHA) Type IV–VIII plaque were designated as Max-diseased tissue segment. Sections of tissue at the peripheral end of the plaque demonstrating AHA type I-III plaques was designated as Min-diseased segments (Figures 1A–D). Min and Max-diseased carotid plaque segments were isolated, partitioned into segments for paraffin embedding, and segments were used to isolate mRNA and protein purification (22). For each patient, Min-diseased segments served as internal controls for the Max-diseased segments. For mRNA isolation, specimens were placed in Trizol (1 mL for 50 mg of tissue) and grinded with mortar and pestle.

Similarly, lower extremity arterial segments were harvested immediately following major lower extremity amputation in the operating room. If available, preoperative imaging was evaluated to determine which lower leg arterial segments were indeed Min or Max-diseased. At least a 5 cm segment of the popliteal artery, anterior tibial, posterior tibial, and/or peroneal arteries were dissected from adjacent tissue in the amputated limb, and arterial segments were placed in cold saline solution and immediately transported to the laboratory on ice. Approximately a 1 cm section of tissue of the most diseased region was designated as Max-diseased while the least diseased segments from the same limb was used as an internal control and designated as Min-diseased (Figures 1E–H). The arterial adventitia was carefully removed from the intima and media layers. Min and Max-diseased arterial segments were embedded in paraffin, or placed in Trizol solution for subsequent mRNA isolation.

### mRNA Isolation and RT-PCR

mRNA was purified from Min and Max-diseased CEA and lower leg arterial segments. All samples were treated with chloroform and centrifuged at 16,000 g at 4°C for 15 min. Supernatants were mixed with an equal volume of 70% ethanol, and centrifuged again at 10,000 g for 1 min. RNA was then isolated from the supernatant using a Qiagen RNA mini-column isolation kit (74104, Qiagen, Venlo, Netherlands). The extracted RNA mixture was amplified using SYBR Green PCR Master Mix (4309155, Thermo Fisher) and a 7500 Fast Real-Time PCR system (Applied Bio-systems, Carlsbad, CA, United States). mRNA primers (Supplementary Table III) were used to evaluate the relative expression of *rpl32, ppara, acox1* and *cpt1a*. Values were calculated using the 2^–ΔΔ*CT*^ method, and normalized relative to the abundance of *rpl32.* The normalized *rpl32* levels were based on abundance of gene expression in the Min-diseased samples from patients with no diabetes.

### Western Blot Analysis

Carotid endarterectomy and lower extremity tissue were also homogenized in cold mammalian cell lysis kit (MCL1-KT; Millipore Sigma). Total protein for both Min and Max-diseased was determined using Bradford Protein Assay and loaded onto Bis-Tris gel (NW00082BOX; Thermo Fisher Scientific) and transferred to polyvinylidene fluoride membranes for Western blotting. Protein was detected with mouse anti-PPARα (SAB4502260; Millipore Sigma), mouse anti-ACOX1 (sc-517306, Santa Cruz), and mouse anti-CPT1a (66039–1-Ig; Proteintech). Rabbit anti-beta actin (ab8227, Abcam) or rabbit anti-GAPDH (G9545, Sigma) was used for Western blot loading controls. Band densitometry analysis was performed using ImageJ software as previously described (27). Band densities were expressed as ratios relative to the protein loading control.

### Tissue Histology

Following collection from the operating room, arterial samples were then prepared as previously described (28). Briefly, Min or Max-diseased arterial samples were sectioned into 3 mm sections, fixed in 10% formalin and decalcified in EDTA for 2 weeks. Samples were washed with 70% ethanol and embedded into paraffin blocks. Paraffin samples were cut into 10 μm sections using a HM324 Rotary Microtome (902100A, ThermoFisher Scientific, Waltham, MA, United States). Slides were stained with pentachrome staining (KTRMP, StatLab, McKinney, TX, United States). Images of stained arterial segments were captured with a NanoZoomer 2.0-HT (Hamamatsu Photonics, K.K., Japan).

### Statistical Analysis

Non-parametric two-tailed Man-Whitney tests were used to evaluate the differences of mRNA expression between diabetic and non-diabetic patients, and non-parametric two tailed Wilcoxon matched-pairs test were used to compare Min and Max samples. Additionally, Kruskal-Wallis Tests and Dunn’s multiple comparisons tests were used to measure differences in mRNA and protein expression between Min and Max diseased segments of patients with and without diabetes. Non-parametric Spearman correlations were calculated comparing the mRNA 2^–ΔΔ*CT*^ values and relative mRNA levels to patient demographics. Outliers were determined by ROUT test and not included in analyses. All analyses were performed using GraphPad Prism (GraphPad Prism 9.1 software, GraphPad Software Inc., United States). We considered *p* < 0.05 to be significant. Data are presented as mean ± SD.

## Results

### *ppara*-Related Gene Expression and Relative Protein Content in Carotid Endarterectomy Plaques

We evaluated *ppara*, and its downstream genes *acox1* and *cpt1a* in Min and Max-diseased CEA plaques from patients with or without diabetes. Although we observed no overall difference in gene expression between Min and Max CEA plaque segments (Figures 2A–C), there was higher *ppara* and *acox1* in CEA plaques of patients with diabetes (*p* < 0.01, *p* < 0.001, respectively; Figures 2D–H). No difference in *cpt1a* expression was observed (Figure 2F). When samples were stratified by diabetes status and disease severity, Min were observed to have higher *ppara* (*p* < 0.05; Figure 2G) compared to Max in individuals with diabetes. There was no difference in *acox1* or *cpt1a* relative to diabetes status or disease severity (Figures 2H,I), and no differences between Min and Max relative to high or low DCSI scores (Supplementary Figure 1).

**FIGURE 3:**
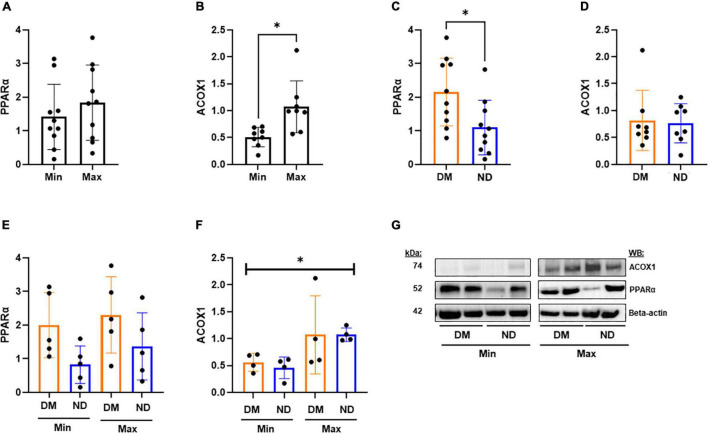
Western blot analysis from CEA in varying disease and diabetic status. **(A,B)** We observed no significant difference in PPARα protein expression between Min (*n* = 10) and Max (*n* = 10) segments, but a significant decrease in relative ACOX1 abundance in Min tissues compared to Max (*p* < 0.05). **(C)** Patients with diabetes (*n* = 10) demonstrate significantly higher relative PPARα abundance compared to patients without diabetes (*p* < 0.05). **(D)** No significant difference in ACOX1 expression among patients with an without diabetes. **(E)** No significant variability in PPARα from combined analysis of diabetic status and lesion severity analysis. **(F)** Kruskal-Wallis test shows significant variability in relative ACOX1 abundance between Min and Max specimens from patients with (*n* = 4) or without (*n* = 5) diabetes. **(G)** Representative Western Blots from analysis. Samples were normalized to beta-actin content. DM, Diabetic. ND, Non-diabetic. Min, Minimally diseased. Max, Maximally diseased. Data are presented as Mean ± SD, and each point represents one patient sample. *p < 0.05.

We evaluated relative protein abundance to determine whether differences in gene expression translated into differences in PPARα downstream signaling in CEA tissue (Figure 3). Although no difference was observed in PPARα between Min and Max, there was higher ACOX1 in Max segments (*p* < 0.05; Figures 3A,B). Consistent with gene expression, we observed higher PPARα among patients with diabetes (*p* < 0.05; Figure 3C). A combined analysis of diabetes status and disease severity showed no difference in PPARα (Figure 3E). However, a combined analysis demonstrated significant variability in relative ACOX1 abundance by ANOVA (*p* < 0.05, Figure 3F), and a non-significant increase in Max samples compared to Min samples in patients with diabetes (Figure 3F, *p* = 0.07) as demonstrated by western blot analysis (Figure 3G).

### Correlation of *ppara*-Related Gene Expression in Carotid Endarterectomy Plaques With Hemoglobin A1c and Serum Glycemic Profiles

**FIGURE 4:**
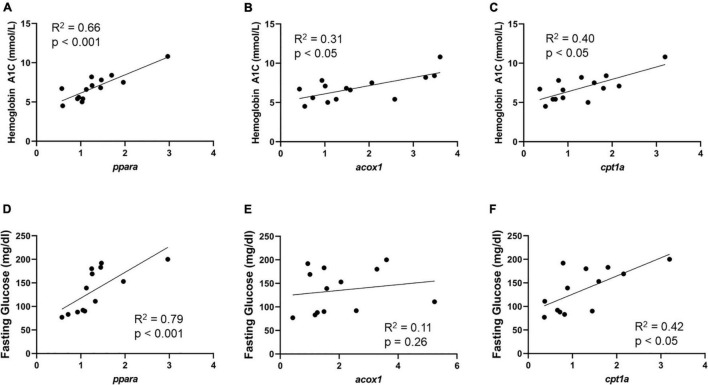
Min carotid plaque mRNA correlates with HbA1c, and fasting glucose content. **(A–C)** Min-diseased carotid plaque mRNA content demonstrates significant positive correlation between *ppara* (*p* < 0.0001, R^2^ = 0.66, *n* = 14), *acox1* (*p* < 0.05, R^2^ = 0.31, *n* = 14) and *cpt1a* (*p* < 0.05, R^2^ = 0.40, *n* = 14) in comparison to HbA1c content. **(D–F)** Min-diseased carotid plaque *ppara* (*p* < 0.001, R^2^ = 0.79, *n* = 13), and *cpt1a* (*p* < 0.05, R^2^ = 0.42, *n* = 13) Demonstrates significant positive correlation with fasting glucose levels. We did not observe a correlation between *acox1* (*p* = 0.26, R^2^ = 0.12, *n* = 13) and fasting glucose.

Gene expression in Min and Max-diseased CEA plaque segments were evaluated relative to hemoglobin A1c and serum fasting glucose (Supplementary Table I). In Min-diseased CEA plaque segments we observed a significant positive correlation between hemoglobin A1c and *ppara* (R^2^ = 0.66, *p* < 0.001), *acox1* (R^2^ = 0.31, *p* < 0.05), and *cpt1a* (R^2^ = 0.40, *p* < 0.05). In Max-diseased plaque segments there was also a significant correlation between hemoglobin A1c and *ppara* (R^2^ = 0.32, *p* < 0.05, Supplementary Figure 2A) and *acox1* (R^2^ = 0.45, *p* < 0.05, Supplementary Figure 2B) expression, but no significant correlation in relative *cpt1a* mRNA expression (Supplementary Figure 2C). We also observed a significant correlation between fasting glucose and *ppara* gene expression in Min diseased plaques (R^2^ = 0.79, *p* < 0.001; Figure 4D), and *cpt1a* (R^2^ = 0.42, *p* < 0.05; Figure 4F), as well as a significant correlation between fasting glucose and *ppara* in Max diseased plaques (R^2^ = 0.38, *p* < 0.05, Supplementary Figure 2D). No correlations were observed with hemoglobin A1C (Figures 4A–C), and we observed no correlation between fasting glucose and *acox1* in Min (Figure 4E) and *acox1* or *cpt1a* Max diseased plaques (Supplementary Figures 2D–F).

Correlation between gene expression in CEA plaque segments and triglyceride, total cholesterol, LDL, HDL, non-HDL cholesterol, and DCSI score were performed. With the exception to moderate positive correlation between *cpt1a* and triglyceride in Min segments (R^2^ = 0.37, *p* < 0.05; Supplementary Table I), and a negative correlation of DCSI scores to *cpt1a* in Max segments (R^2^ = 0.54, *p* < 0.05, Supplementary Table I), no other correlations were observed (Supplementary Table I).

### *ppara*-Related Gene Expression and Relative Protein Abundance in Lower Extremity Arterial Segments

**FIGURE 5:**
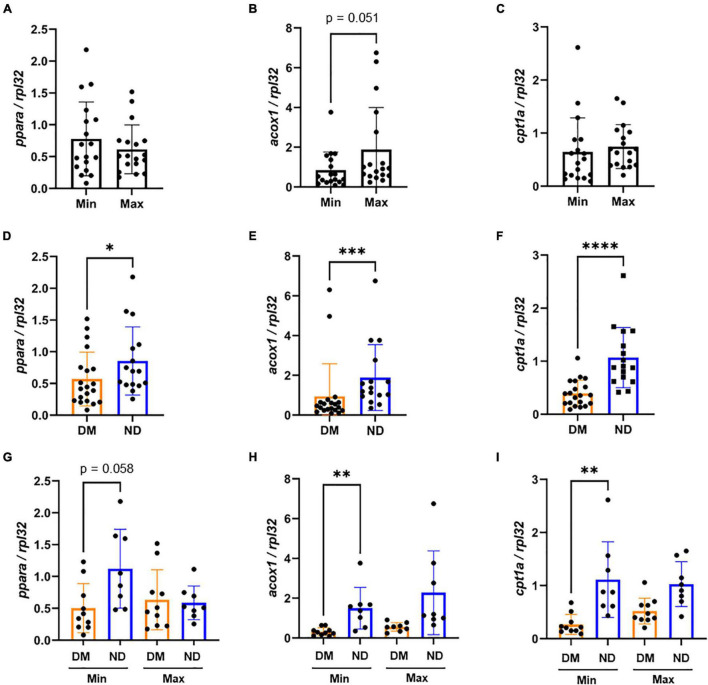
mRNA expression variability of lower extremity segments by lesion severity and diabetic status: **(A–C)** In Lower extremity samples, we observed no significant difference in *ppara* and *cpt1a* mRNA expression between Min (*n* = 18) and Max (*n* = 18) diseased segments. However, we found Min-diseased samples had non-significantly lower expression of *acox1* (*p* = 0.051, *n* = 18) compared to Max-diseased. **(D–F)** We found that patients with diabetes (*n* = 20) demonstrated significantly lower *ppara* (*p* < 0.05), *acox1* (*p* < 0.001) and *cpt1a* (*p* < 0.0001) mRNA expression compared to patients without diabetes (*n* = 16). **(G–I)** When categorizing segments between diseased and diabetic status, we observed no significant variability of *ppara*. However, we found a significant decrease of *acox1* (*p* < 0.01) and *cpt1a* (*p* < 0.01) in patients with diabetes (*n* = 10) compared to patients without diabetes (*n* = 8) from Min-diseased tissues. DM, Diabetic. ND, Non-diabetic. Min, minimally diseased. Max, maximally diseased. Data are presented as Mean ± SD and, each point represents one patient sample. *p < 0.05, **p < 0.01, and ***p < 0.001.

Expression of *ppara, acox1*, and *cpt1a* was evaluated in Min and Max-diseased lower extremity arterial segments in individuals with or without diabetes. Similar to findings in CEA plaques, we observed no differences in *ppara*, and *cpt1a* in Min-diseased lower extremity arterial segments, but 44% lower *acox1* expression (*p* = 0.05; Figures 5A–C). Unlike CEA plaque, there was lower *ppara* (*p* < 0.05), *acox1* (*p* < 0.001) and *cpt1a* (*p* < 0.0001) expression in lower extremity segments from patients with diabetes (Figures 5D–F). Combined analysis of arterial disease severity and diabetes status demonstrated no difference in *ppara* (Figure 5G), but significant variability among in *acox1* (*p* < 0.01; Figure 5H) and *cpt1a* expression (*p* < 0.01; Figure 5I) particularly in Min segments. Unlike individuals with low DCSI scores, we observed that individuals with high DCSI scores had higher relative *acox1* (*p* < 0.05) and *cpt1a* (*p* < 0.05) expression in Max segments (Supplementary Figure 3).

**FIGURE 6:**
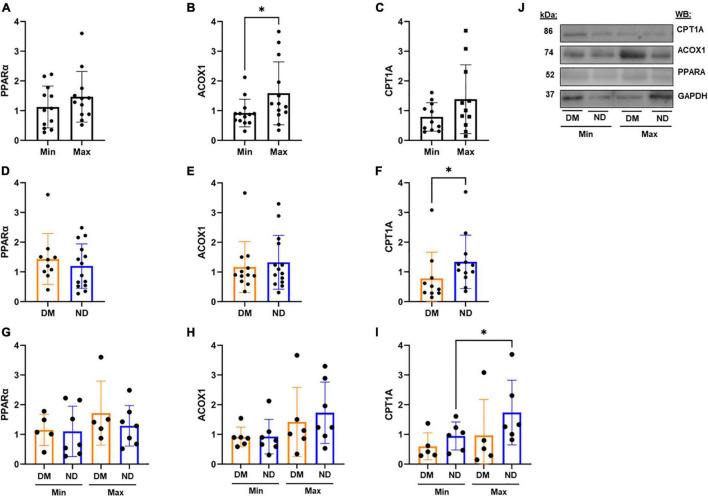
Western Blot Analysis from Lower Extremity Segments in Varying Disease and Diabetic Status. **(A–C)** In lower extremity arterial segments, we found no difference in relative PPARα (*n* = 12) or CPT1A (*n* = 11) abundance between Min or Max segments. However, we found Min segments demonstrate a decrease in ACOX1 compared to Max samples (*p* < 0.05, *n* = 13). **(D–F)** We found no difference in relative PPARα abundance between patients with and without diabetes. ACOX1 demonstrated decreased expression in patients with diabetes (*n* = 13) compared to patients without diabetes (*n* = 13), and CPT1A similarly showed a decrease in expression in patients with diabetes (*n* = 10) compared to patients without (*n* = 12). **(G–I)** Subdividing tissue segments into both disease and diabetic status showed no significant variability in PPARα or ACOX1 expression. However, CPT1A expression was decreased in Min samples compared to Max samples in patients without diabetes (*n* = 6). **(J)** Representative Western Blots from analysis. Samples were normalized to GAPDH content. DM, Diabetic. ND, Non-diabetic. Min, Minimally diseased. Max, Maximally diseased. Data are presented as Mean ± SD, and each point represents one patient sample. *p < 0.05.

There was no difference in relative PPARα or CPT1A protein abundance between Min or Max-diseased lower extremity arterial segments, but there was significant lower ACOX1 in Min segments (*p* < 0.05, Figures 6A–C). While no difference in PPARα or ACOX1 was observed in individuals with and without diabetes, individuals with diabetes had higher CPT1A (*p* < 0.05) compared to those without diabetes (Figures 6D–F). A combined analysis of lesion severity and diabetes status found no difference in PPARα, or ACOX1, but a significant increase of CPT1A (*p* < 0.05) in Max segments in individuals without diabetes (Figures 6G–I) as demonstrated by western blot analysis (Figure 6J).

### Lower Extremity *ppara* Signaling Correlates With Hyperlipidemic Markers

**FIGURE 7:**
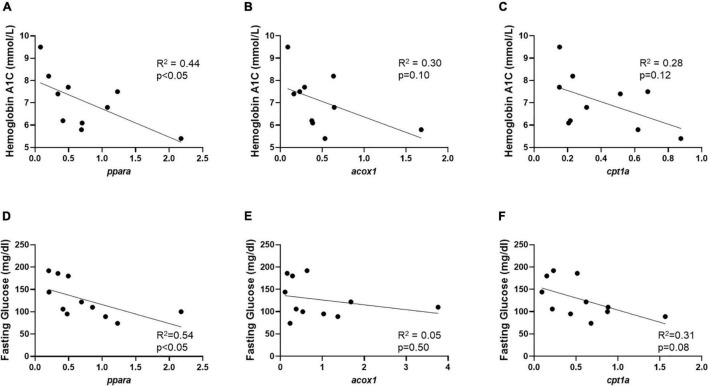
Min lower extremity mRNA correlates with HbA1c and fasting glucose content. **(A)** Min-diseased lower extremity segment relative mRNA content demonstrates significant negative correlation between *ppara* (*p* < 0.05, R^2^ = 0.44, *n* = 10) compared to HbA1c content. **(B,C)** No significant correlation was observed between *acox1* (*p* = 0.10, R^2^ = 0.30, *n* = 10) and *cpt1a* (*p* = 0.12, R^2^ = 0.28, *n* = 10) relative mRNA abundance compared to HbA1c content in Min-diseased lower extremity segments. **(D)** Min-diseased lower extremity segment *ppara* (*p* < 0.05, R^2^ = 0.54, *n* = 11) relative mRNA abundance shows significant correlation with fasting glucose. **(E,F)** No significant correlation was observed between fasting glucose and *axoc1* (*p* = 0.50, R^2^ = 0.05, *n* = 11) or *cpt1a* (*p* = 0.08, R^2^ = 0.31, *n* = 11) relative mRNA abundance.

Similar to analysis in CEA segments, we evaluated the correlation of lower extremity arterial segment gene expression and serum glycemia and lipid profiles (Supplementary Table II). Unlike findings in CEA, we observed a negative correlation between *ppara* and hemoglobin A1c (R^2^ = 0.44, *p* < 0.05), fasting glucose (R^2^ = 0.54, *p* < 0.05; Figure 7), and a positive correlation with HDL (R^2^ = 0.42, *p* < 0.05; Supplementary Table II). *acox1* in lower extremity Min segments has a positive correlation with total cholesterol as well as HDL (R^2^ = 0.42, *p* < 0.05; R^2^ = 0.60, *p* < 0.05; respectively; Supplementary Table II). No significant correlation was observed between *cpt1a* from Min segments, or *ppara, acox1*, and *cpt1a* from Max segments (Supplementary Table II).

## Discussion

This study provides novel findings on *ppara*-related gene expression and relative protein content in peripheral carotid and lower leg arterial segments—two variant anatomical regions that are prone to high burden of atherosclerotic disease progression in patients with cardiovascular risk factors such as diabetes and hyperlipidemia (11–14). We observed that *ppara* and its downstream genes, *acox1* and *cpt1a*, indeed have significant variable expression between individuals with and without diabetes in both carotid and lower leg arterial tissue. In particular, Min tissue from CEA plaques and lower extremities both demonstrated lower ACOX1. Interestingly, in CEA tissue, we observed that PPARα was significantly higher in patients with diabetes, and had a significant positive correlation with hemoglobin A1c and fasting glucose. PPARα is a key transcription factor for downstream genes such as *acox1* and *cpt1a*, which both also significantly correlated with hemoglobin A1c levels. On the other hand, in lower extremity arterial segments, *ppara, acox1*, and *cpt1a* all had lower expression in individuals with diabetes. CPT1A was also lower in lower extremity arterial tissue from individuals with diabetes. Furthermore, Min segments demonstrated a negative correlation between *ppara* and hemoglobin A1c. Taken together these findings demonstrate that in the setting of diabetes, *ppara* expression and signaling is highly variable in different arterial beds—particularly in the carotid and lower leg arterial systems. These observations may have important implications in understanding risk factors for atheroprogression in these anatomical regions, as well as the impact of current PPARα-targeting pharmacological therapy.

PPARα is a major regulator of lipid metabolism, serum triglycerides, and peripheral atherosclerosis (18, 20, 21, 28). It is expressed as a nuclear membrane protein in metabolic tissue that utilizes β-fatty acid oxidation for cellular signaling and homeostasis (29–31). In organ tissue such as liver, kidney, heart, and muscle, PPARα is activated by lipid precursors such as phosphatidylcholines (PCs), and forms a heterodimer with retinoid X receptor (RXR) to activate downstream target genes such as *acox1* and *cpt1a* (17, 32–36). While PPARα has hundreds of downstream targets, upregulation of *acox1* is critically important for peroxisomal fatty acid beta-oxidation that breaks down very long-chain fatty acids (34, 37). Similarly, PPARα activates *cpt1a* which is responsible for attaching carnitine subgroups to long-chain fatty acids so that they can be metabolized in the mitochondria to produce ATP (38). While PPARα expression is thought to modulate inflammation and reduce atherosclerosis in the vessel wall, it is largely unknown what roles its downstream genes *acox1* and *cpt1a* play in accumulating saturated fatty acids and contribute to atheroprogression in carotid and lower extremity arterial distributions (18–21).

Previous studies have evaluated the impact of targeting PPARα on atheroprogression. For example, the FIELD study is a multi-center, prospective, randomized clinical trial that evaluated the impact of fenofibrate, a PPARα-agonist, on cardiovascular outcomes in individuals with diabetes (39). The study observed no difference in primary composite endpoints of coronary artery disease, death, and non-fatal myocardial infarction (MI). However, the study secondary composite endpoint of total cardiovascular disease events were reduced in patients who were randomized to fenofibrate. In fact, the study uniquely demonstrated that treatment with fenofibrate can decrease the risk of lower extremity amputations, particularly minor amputations without known large-vessel inflow arterial obstruction in the upper leg. These findings suggested that fenofibrate can potentially impact atheroprogression in lower extremity arterial beds. Several subsequent studies have argued that the impact of fenofibrate on the peripheral arterial tissue may be occurring though non-lipid mechanisms (40, 41), and determining the impact of fenofibrate on peripheral arterial tissue remains a topic of investigation.

Similar studies have also evaluated the impact of PPARα signaling in carotid atheroprogression. A within-gene haplotype analysis demonstrated that *ppara* was one of 10 genes that impacts inflammation and endothelial function in individuals with dense carotid plaque as demonstrated on ultrasound B-mode imaging (42). Genotyping of genetic variants in > 3,300 individuals from the Framingham Offspring Study also demonstrated that a *ppara* genetic variant (L162V) was associated with incidence of carotid artery stenosis (43). Pharmacological targeting of PPARα with fenofibrate in pre-clinical models has demonstrated atherosclerotic plaque regression in the setting of hyperlipidemia (44). In human subjects, fenofibrate has also been linked to reduced carotid intima-media thickness in a subpopulation of individuals with diabetes in the FIELD study, highlighting the systemic benefits of reduced hypertriglyceridemia, as well as the potential benefits of reducing carotid atheroprogression (45). Our study builds on this, and examines whether *ppara* and its downstream genes are differentially expressed relative to disease severity and diabetes status.

Our study observed different gene expression patters of *ppara*, *acox1*, and *cpt1a* in carotid and lower extremity arterial tissue, suggesting that atherosclerotic disease progression is unlikely homogeneous. We similarly observed that other critical lipid mediators are differentially expressed and activated in carotid and lower extremity arterial tissue (23, 46, 47). For example, choline-ethanolamine phosphotransferase 1 (CEPT1), a key regulator of *de novo* phospholipogenesis, is highly expressed in Min-diseased carotid arterial segments particularly in individuals with diabetes (22). Here we observed similar patterns in Min-diseased CEA segments from individuals with diabetes, which demonstrated higher relative *ppara, acox1*, and *cpt1a* mRNA abundance. Taken in context with our previous observations demonstrating increased pro-inflammatory phospholipid content in Min diseased CEA segments, altered beta-fatty acid oxidation signaling via *ppara* may be contributing to endothelial dysfunction, inflammation, and foam cell development—leading to further atheroprogression in at risk populations (48–51). Ongoing studies are evaluating whether similar gene expression patterns are also evident in individuals with other risk factors such as age and smoking habits.

In the lower extremity, we observed totally different gene expression patterns and correlations. Specifically, we observed lower relative *ppara*, *acox1*, and *cpt1a* mRNA abundance from patients with diabetes, and a negative correlation between *ppara* mRNA abundance, hemoglobin A1c, and fasting glucose. These findings are striking given their clear divergence from observations on CEA tissues. These findings suggest that in the setting of diabetes arterial beds vary in β-fatty acid oxidation metabolism particularly in lower extremities compared to carotid arteries. We previously observed similar expression patterns in CEPT1 metabolism in Max-diseased lower extremity arterial segments, suggesting that arterial lipid metabolism is significantly impacted by disease progression and endothelial dysfunction in these segments (23). Interestingly, our current study did not observe that DCSI score was indicative of altered β-fatty acid oxidation. We suspect that this may be either due to the multi-factorial DCSI scoring algorithm that is not entirely based on severity of peripheral arterial disease, or may be due to lack of statistical power in the current analysis. Ongoing investigation in larger populations, and utilization of additional surrogate measurements for peripheral arterial disease severity, will help further clarify to what extent *ppara* gene expression is impacted by atherosclerotic disease severity in the setting of diabetes.

We acknowledge that our current study has some limitations. First, the limited number of patients could have led to some sampling errors, and may not have fully accounted for the range of co-morbidities that can impact carotid and lower extremity atheroprogression. Ongoing studies are prospectively enrolling larger patient populations to validate our reported study findings. Second, due to the number of recruited patients, we were not able to fully match gene expression analysis by age, co-morbidities, and sex demographics. It is possible that such demographics, in addition to smoking history and variation in lipid parameters (total cholesterol, LDL, and HDL), could have impacted our study results. Third, RNA analysis with RTPCR was limited to CEA or lower leg arterial segments, and did not differentiate cell types with in the tissue specimens. Due to the severity of occlusive disease in patients undergoing lower extremity amputation, harvesting a completely “healthy” sample as a control for analysis was not possible. Alternatively, Min-diseased segments were routinely harvested and served as an internal patient control for gene expression analysis. Finally, despite careful tissue sample dissection, we acknowledge that carotid and lower extremity arterial tissue represents a heterogenous cellular phenotype. Future analysis with single-cell techniques can help further delineate expression patterns across unique cell types of interest.

In conclusion, we report novel findings on the variable *ppara* gene expression in Min vs. Max CEA and lower extremity arterial segments. We also demonstrate that PPARα downstream genes *acox1* and *cpt1a* have different expression patterns relative to tissue type, arterial disease severity, and patient diabetes status. We postulate that differences in relative *ppara* expression and downstream activation may impact arterial atheroprogression and have important implications on future pharmacotherapy options.

## Data Availability Statement

The original contributions presented in the study are included in the article/Supplementary Material, further inquiries can be directed to the corresponding author.

## Ethics Statement

The studies involving human participants were reviewed and approved by Washington University School of Medicine IRB. The patients/participants provided their written informed consent to participate in this study.

## Author Contributions

CE, NH, GD, CS, and MZ: conception and design. CE, NH, RM, AP, XJ, CS, and MZ: analysis and interpretation. CE, NH, RM, AP, and MS: data collection. CE and MZ: writing the manuscript. CE, NH, RM, AP, MS, XJ, GD, CS, and MZ: critical revision and final approval of the manuscript. CE, AP, and XJ: statistical analysis. MZ: obtained funding and overall responsibility. All authors contributed to the article and approved the submitted version.

## Conflict of Interest

The authors declare that the research was conducted in the absence of any commercial or financial relationships that could be construed as a potential conflict of interest.

## Publisher’s Note

All claims expressed in this article are solely those of the authors and do not necessarily represent those of their affiliated organizations, or those of the publisher, the editors and the reviewers. Any product that may be evaluated in this article, or claim that may be made by its manufacturer, is not guaranteed or endorsed by the publisher.
