# Supplementary material for: Altered Peroxisome Proliferator-Activated Receptor Alpha Signaling in Variably Diseased Peripheral Arterial Segments
Source: Front Cardiovasc Med. 2022 Jun 15;9:834199. doi: 10.3389/fcvm.2022.834199 (PMC9248745; doi:10.3389/fcvm.2022.834199)
Supplement: Supplementary file 1 [file Table_1.DOCX]

Supplementary Material


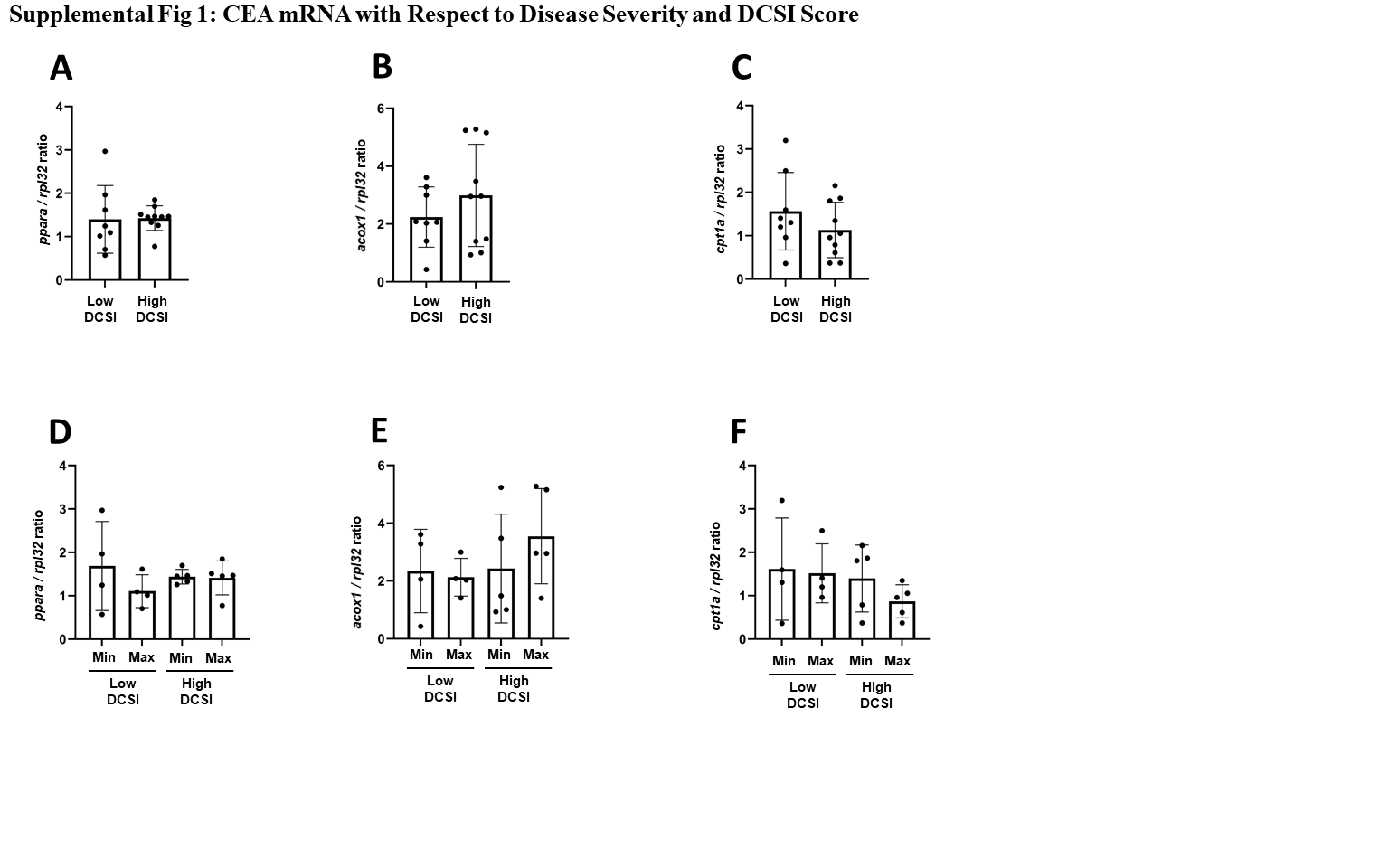


**Supplemental Figure 1: CEA mRNA with Respect to Disease Severity and DCSI Score.** **A-C:** We observed no significant difference between the relative abundance of *ppara, acox1* or *cpt1a* mRNA between patients with high (n=8) or low (n=10) DCSI scores in carotid endarterectomy plaques. **D-F:** When distinguishing both DCSI score and disease severity, we similarly observed no variability in mRNA expression between patients with low DCSI scores (n=4) in Min or Max segments and patients with high DCSI scores (n=5) in Min or Max segments. The mean DCSI score of all carotid patients and was used to separate patients into the ‘high’ or ‘low’ category. Patient with a score below 4 were assigned to the ‘low’ group while patients with a score of 4 or more was assigned to the ‘high’ group. Error bars represent SD.


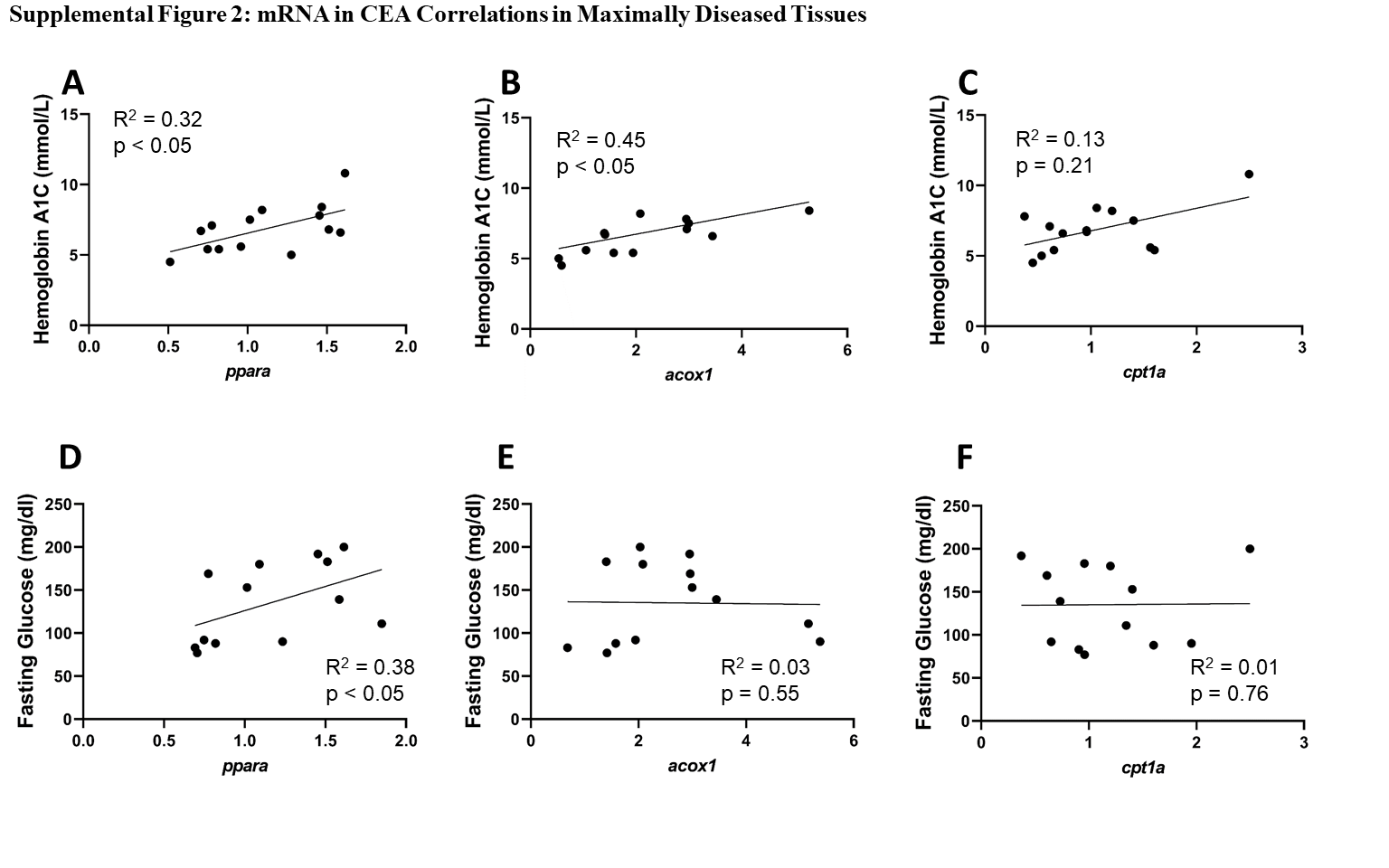


**Supplemental Figure 2: mRNA in CEA Correlations in Maximally Diseased Tissues. A-C:** Max-diseased carotid plaque mRNA demonstrates significant correlation between *ppara* (p<0.05, n=14), *acox1* (p<0.05 n=14) and *cpt1a* (p<0.05, n=14) in comparison to HbA1c content. **D-F:** Max-diseased carotid plaque mRNA demonstrates significant correlation between *ppara* (p<0.05, n=13) with fasting glucose concentration. *acox1* and *cpt1a* did not demonstrate a significant trend.


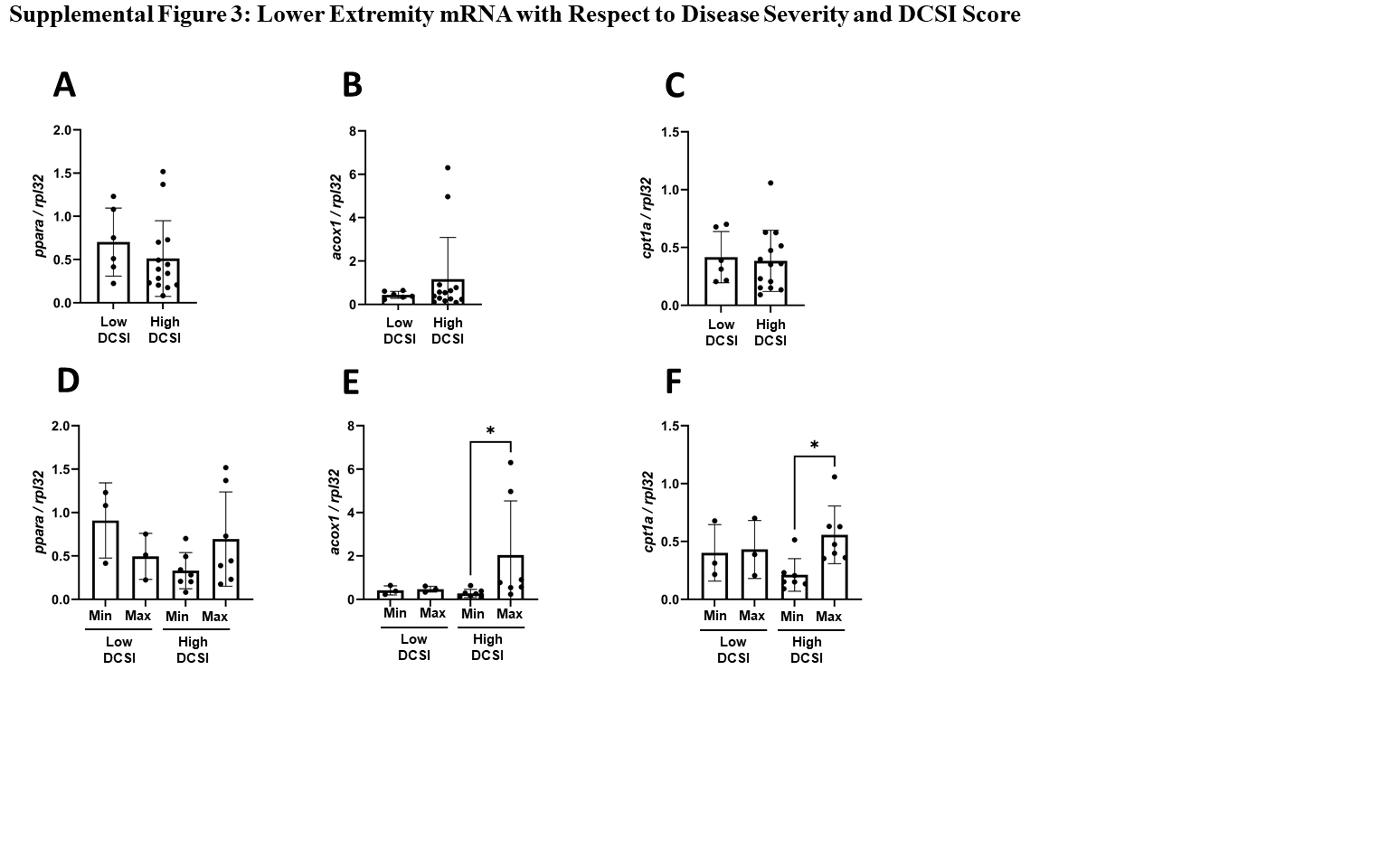


**Supplemental Figure 3: Lower Extremity mRNA with Respect to Disease Severity and DCSI Score.** **A-C:** We observed no significant difference between *ppara, acox1* or *cpt1a* mRNA between patients with high (n=6) or low (n=14) DCSI scores in carotid endarterectomy plaques. **D:** No significant difference in relative *ppara* abundance was observed between patients with low DCSI scores (n=3) or High DSCI scores in Min or Max tissues. **E&F:** We found a significant increase in relative *acox1* and *cpt1a* abundance between Min and Max segments in High DCSI scoring patients (p<0.05, n=7). The mean DCSI score of all amputation patients and was used to separate patients into the ‘high’ or ‘low’ category. Patient with a score below 6 were assigned to the ‘low’ group while patients with a score of 6 or more was assigned to the ‘high’ group. Min, Minimally diseased. Max, Maximally diseased. Error bars represent SD, *p < 0.05.


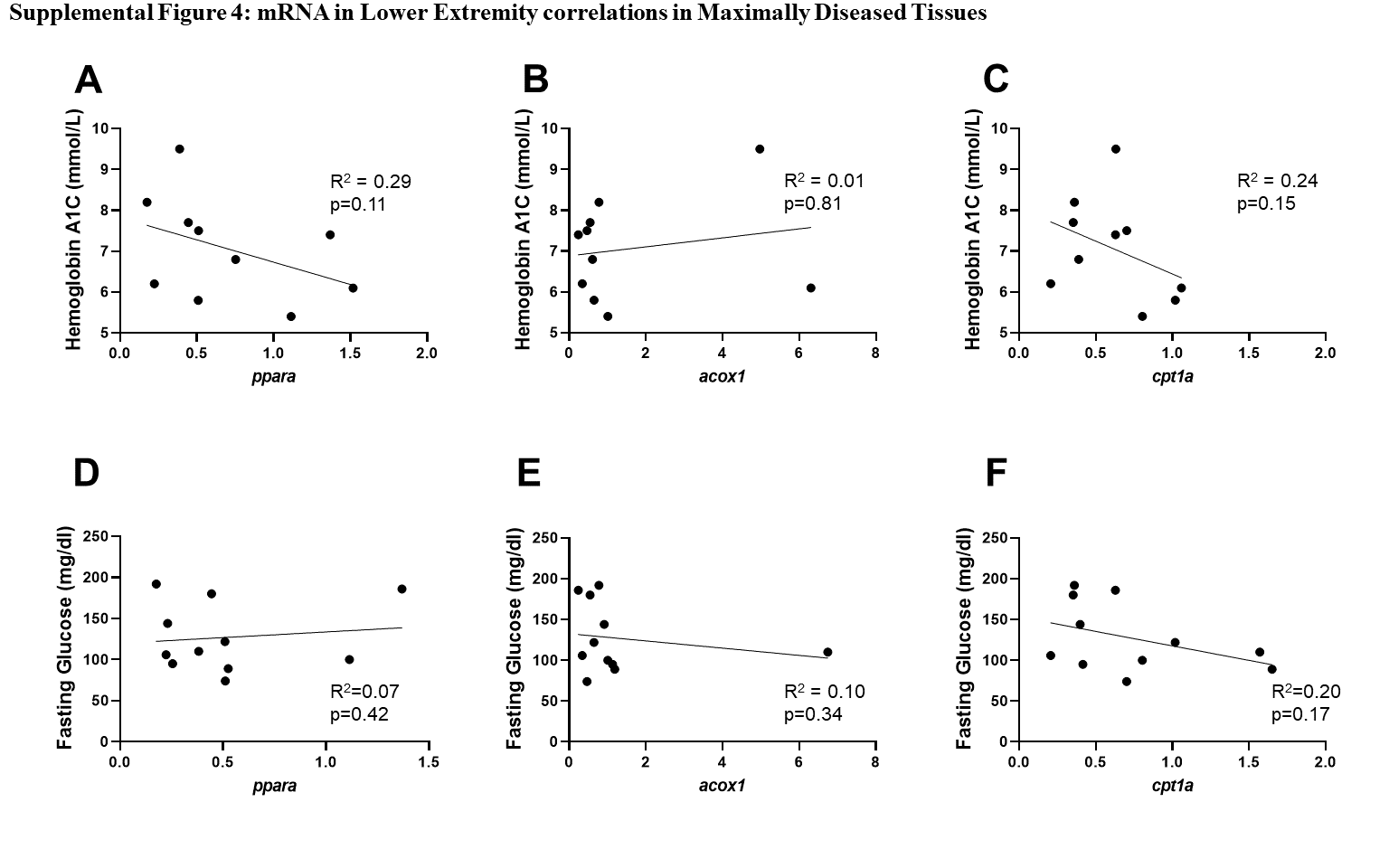


**Supplemental Figure 4**: **mRNA in Lower Extremity Correlations in Maximally Diseased Tissues:** A-C: No significant correlation between Max-diseased lower extremity segments Hemoglobin A1C content and *ppara, acox1,* or *cpt1a* (n=11). D-F: No significant correlation between Max-diseased lower extremity fasting glucose content in *ppara*, *acox1,* or *cpt1a* (n=10).

**Supplemental Table I: Carotid Plaque mRNA Expression and Demographics Correlation**

|  |  | Hemoglobin A1c | Fasting Glucose (mg/dl) | Triglycerides (mg/dl) | Cholesterol (mg/dl) | LDL-cholesterol (mg/dl) | HDL-Cholesterol (mg/dl) | Non-HDL-Cholesterol (mg/dl) | DCSI |
| --- | --- | --- | --- | --- | --- | --- | --- | --- | --- |
| Min *ppara* | r | 0.81 | 0.89 | 0.40 | 0.03 | -0.15 | 0.14 | 0.07 | -0.16 |
|  | R^2^ | 0.66 | 0.79 | 0.16 | 0.00 | 0.02 | 0.02 | 0.00 | 0.03 |
|  | p | **<0.001** | **<0.001** | 0.17 | 0.92 | 0.62 | 0.66 | 0.83 | 0.68 |
| Min *acox1* | r | 0.56 | 0.34 | 0.35 | 0.00 | -0.34 | -0.44 | 0.22 | -0.25 |
|  | R^2^ | 0.31 | 0.11 | 0.12 | 0.00 | 0.11 | 0.19 | 0.05 | 0.06 |
|  | p | **<0.05** | 0.26 | 0.24 | 1.00 | 0.26 | 0.13 | 0.47 | 0.52 |
| Min *cpt1a* | r | 0.63 | 0.65 | 0.61 | 0.09 | -0.09 | -0.14 | 0.24 | 0.07 |
|  | R^2^ | 0.40 | 0.42 | 0.37 | 0.01 | 0.01 | 0.02 | 0.06 | 0.00 |
|  | p | **<0.05** | **<0.05** | **<0.05** | 0.77 | 0.76 | 0.65 | 0.44 | 0.87 |
| Max *ppara* | r | 0.57 | 0.62 | 0.48 | -0.29 | -0.42 | -0.20 | -0.15 | -0.03 |
|  | R^2^ | 0.32 | 0.38 | 0.23 | 0.08 | 0.17 | 0.04 | 0.02 | 0.00 |
|  | p | **<0.05** | **<0.05** | 0.10 | 0.34 | 0.16 | 0.51 | 0.62 | 0.96 |
| Max *acox1* | r | 0.67 | 0.18 | 0.35 | 0.03 | -0.17 | 0.06 | 0.05 | 0.39 |
|  | R^2^ | 0.45 | 0.03 | 0.12 | 0.00 | 0.03 | 0.00 | 0.00 | 0.15 |
|  | p | **<0.05** | 0.55 | 0.24 | 0.93 | 0.58 | 0.85 | 0.86 | 0.29 |
| Max *cpt1a* | r | 0.36 | -0.09 | 0.31 | -0.31 | -0.41 | -0.50 | -0.04 | -0.73 |
|  | R^2^ | 0.13 | 0.01 | 0.10 | 0.09 | 0.17 | 0.25 | 0.00 | 0.54 |
|  | p | 0.21 | 0.76 | 0.30 | 0.31 | 0.17 | 0.08 | 0.91 | **<0.05** |

Min, Minimally diseased. Max, Maximally diseased.

|  |  | Hemoglobin A1c | Fasting Glucose (mg/dl) | Triglycerides (mg/dl) | Cholesterol (mg/dl) | LDL-cholesterol (mg/dl) | HDL-Cholesterol (mg/dl) | Non-HDL-Cholesterol (mg/dl) | DCSI |
| --- | --- | --- | --- | --- | --- | --- | --- | --- | --- |
| Min *ppara* | r | -0.66 | -0.74 | -0.55 | 0.10 | 0.47 | 0.64 | -0.03 | -0.64 |
|  | R^2^ | 0.44 | 0.54 | 0.30 | 0.01 | 0.22 | 0.42 | 0.00 | 0.41 |
|  | p | **<0.05** | **<0.05** | 0.10 | 0.79 | 0.17 | **<0.05** | 0.95 | 0.07 |
| Min *acox1* | r | -0.55 | -0.23 | -0.03 | 0.65 | 0.13 | 0.77 | 0.33 | -0.53 |
|  | R^2^ | 0.30 | 0.05 | 0.00 | 0.42 | 0.02 | 0.60 | 0.11 | 0.28 |
|  | p | 0.10 | 0.50 | 0.95 | **<0.05** | 0.71 | **<0.05** | 0.35 | 0.15 |
| Min *cpt1a* | r | -0.53 | -0.55 | -0.26 | 0.48 | 0.57 | 0.55 | 0.35 | -0.55 |
|  | R^2^ | 0.28 | 0.31 | 0.07 | 0.23 | 0.33 | 0.30 | 0.12 | 0.30 |
|  | p | 0.12 | 0.08 | 0.47 | 0.17 | 0.09 | 0.11 | 0.33 | 0.13 |
| Max *ppara* | r | -0.54 | -0.27 | -0.55 | -0.12 | 0.33 | 0.53 | -0.27 | 0.12 |
|  | R^2^ | 0.29 | 0.07 | 0.30 | 0.01 | 0.11 | 0.28 | 0.07 | 0.01 |
|  | p | 0.11 | 0.42 | 0.10 | 0.76 | 0.35 | 0.12 | 0.45 | 0.76 |
| Max *acox1* | r | -0.09 | -0.32 | 0.08 | 0.02 | -0.07 | -0.15 | -0.08 | 0.57 |
|  | R^2^ | 0.01 | 0.10 | 0.01 | 0.00 | 0.01 | 0.02 | 0.01 | 0.33 |
|  | p | 0.81 | 0.34 | 0.84 | 0.97 | 0.85 | 0.69 | 0.84 | 0.11 |
| Max *cpt1a* | r | -0.49 | -0.45 | -0.49 | -0.15 | 0.09 | 0.26 | -0.28 | 0.42 |
|  | R^2^ | 0.24 | 0.20 | 0.24 | 0.02 | 0.01 | 0.07 | 0.08 | 0.18 |
|  | p | 0.15 | 0.17 | 0.15 | 0.68 | 0.82 | 0.47 | 0.43 | 0.26 |

**Supplemental Table II: Lower Extremity mRNA Expression and Demographics Correlation.**

Min, Minimally diseased. Max, Maximally diseased.

**Supplemental Table III: Primer list**

| Gene | Nucleotide Sequence |
| --- | --- |
| Hu-RPL32 FW | AGAAGTTCATCCGGCACCAG |
| Hu-RPL32 Rev | CTTGACGTTGTGGACCAGGA |
| hu-PPARa FW | CCCCTCCTCGGTGACTTATC |
| hu-PPARa Rev | CTGCGGTCGCACTTGTCATA |
| hu-Acox1 FW | GTGTGTGGCATGGTGTCCTA |
| hu-Acox1 Rev | TCCAAGCTACCTCCTTGCTT |
| hu-CPT1a FW | AAGTTGGCGTCTGAGAAGCAT |
| hu-CPT1a Rev | AGTCAAACAGCTCCACTTGCT |
